# Longitudinal Association of Dietary Energy Density with Abdominal Obesity among Chinese Adults from CHNS 1993–2018

**DOI:** 10.3390/nu14102151

**Published:** 2022-05-21

**Authors:** Haojie Hu, Lijun Zuo, Xiaoyun Song, Chang Su, Huijun Wang, Bing Zhang, Gangqiang Ding

**Affiliations:** 1 National Institute for Nutrition and Health, Chinese Center for Disease Control and Prevention, Beijing 100050, China; 15732683256@163.com (H.H.); sxydljk@126.com (X.S.); wanghj@ninh.chinacdc.cn (H.W.); zhangbing@chinacdc.cn (B.Z.); dinggq@chinacdc.cn (G.D.); 2 National Health Commission Key Laboratory of Trace Element Nutrition, Beijing 100050, China; 3 Aerospace Information Research Institute, Chinese Academy of Sciences, Beijing 100101, China; zuolj@radi.ac.cn

**Keywords:** dietary energy density, waist circumference, abdominal obesity, multilevel model, Chinese

## Abstract

Few studies have explored the longitudinal association between dietary energy density and waist circumference and abdominal obesity in adults in China. This study aimed to analyze the relationship between dietary energy density and waist circumference and abdominal obesity in Chinese residents aged 18–64. Using data from the CHNS from 1993 to 2018, 25,817 adult residents aged 18 to 64 were selected for the study. Three consecutive 24-h dietary recalls and home-weighed seasonings were used to assess food intake. A multilevel model was used to analyze the relationship between dietary energy density and waist circumference and abdominal obesity. The results showed that compared with the lowest dietary energy density group, females had an increased risk of abdominal obesity (OR = 1.16, 95% CI: 1.05, 1.29), and females’ waist circumference increased significantly by 0.24 cm (95% CI: 0.39–1.09) in the highest dietary energy density group. No association between dietary energy density and waist circumference and abdominal obesity was observed in males. This study shows that higher dietary energy density is significantly associated with females’ waist circumference and abdominal obesity. Further research on high dietary energy density and abdominal obesity will provide scientific basis for the effective control of abdominal obesity.

## 1. Introduction

Abdominal obesity refers to the excessive accumulation of fat in the abdomen, which is a risk factor for metabolic syndrome, type 2 diabetes, and cardiovascular disease [1,2]. The prevalence of abdominal obesity is increasing all over the world. Data from the National Health and Nutrition Examination Survey (NHANES) showed that the age-adjusted prevalence of abdominal obesity in adults aged 20 and over increased from 46.4% in 1999–2000 to 54.2% in 2011–2012 [3]. The age-adjusted prevalence of abdominal obesity among Chinese adults increased from 19.84% in 1993 to 43.15% in 2011 [4]. Excessive energy intake is an important risk factor for the increased incidence of abdominal obesity. The amount of energy intake is affected by dietary energy density (DED) [5]. Since an individual’s daily food intake remains relatively constant for a period of time, eating the same weight of high-energy-density food will lead to excessive energy intake [6].

Energy density (ED) is defined as the energy per unit weight of food (kcal/g). ED includes single food energy density and total food energy density. Only focusing on the energy density of a single food will ignore the interaction between multiple foods, and cannot comprehensively evaluate the diet. Therefore, it is very important to calculate the energy density of the whole diet [7].

Previous studies have mostly focused on the relationship between DED and body mass index (BMI) [8]. However, BMI does not consider the distribution of fat in the body, so it is weaker than waist circumference (WC) in identifying risk factors related to metabolism and cardiovascular disease [9,10]. Regarding whether DED is associated with abdominal obesity in adult residents, the conclusions are inconsistent. The study of Spanish residents showed that the risk of abdominal obesity increased with the increase of DED [11]. The results of studies on Iranian adults aged 18–59 showed that there was no significant correlation between DED and abdominal obesity [12]. There are few studies on the relationship between DED and abdominal obesity in China, and most of them are cross-sectional studies on the relationship between DED and overweight and obesity [5,13]. There are huge differences in the diet and cooking methods between China and other countries, so it is necessary to carry out relevant research on the Chinese population. Using data from the China Health and Nutrition Survey (CHNS, 1993–2018), this study aims to explore the relationship between DED and abdominal obesity and to provide evidence for future dietary management and prevention of abdominal obesity.

## 2. Materials and Methods

### 2.1. Study Population

CHNS is a family-based longitudinal survey launched in 1989. So far, 11 rounds of follow-up surveys have been conducted on the socio-economic status, health services, and dietary structure of the same population from 1989 to 2018. The community was selected using a multi-stage stratified cluster sampling design, and the survey contents included individual, household, and community materials. The detailed background, aims, design, and methods of the CHNS have been described in the published literature [14,15].

Our analysis selected survey data between 1993 and 2018, as waist circumference measurement started in 1993. All the adults (aged 18–64) completed data on socioeconomic status, demographics, and three-day 24 h dietary recalls in a survey year. We excluded participants who reported implausible values, <500 kcal/d or >5000 kcal/d for energy intakes, <300 g/d or >3000 g/d for food consumption, <50 cm or >130 cm for waist circumstance (WC) [16,17]. Our final sample consisted of 25,817 participants (12,193 male and 13,624 female) clustered in 402 communities, who participated in at least one round from 1993 to 2018. There was a total of 70,469 observations across the nine survey years.

The survey was approved by the institutional review committees of the University of North Carolina at Chapel Hill and the National Institute for Nutrition and Health, Chinese Center for Disease Control and Prevention (No. 2019-024). All the participants provided written informed consent.

### 2.2. Dietary Data

The daily dietary intake information of each subject was recalled for three consecutive days, and the condiments of the family were weighed, of which three consecutive days should be two working days and one weekend. Investigators visit households every day to inquire about dietary conditions and complete the dietary survey data collation on the day of the survey. The percentage of the oil, salt, and other condiments from the household inventory that each member consumed by the ratio of their energy intake to the energy intake of all members was determined. The China Food Composition Tables were used to calculate mean daily energy and nutrient intakes from the food consumption data for each individual [18].

### 2.3. Definition of DED and Abdominal Obesity

DED was calculated by dividing the reported mean energy intake by the total grams consumed. DED was calculated excluding beverages and alcohol. Quartiles of DED were created according to the survey year and gender [19]. The grouping criteria of dietary energy density has been described in the Table S1.

The waist circumference was measured on each individual by trained health workers. Waist circumference is measured with Seca-201 inelastic tape. The tape was placed at the midpoint between the lower margin of the arcus costalis on the midaxillary and the interiliac crest lines and was accurate to 0.1 cm. Waist circumference ≥85 cm for men and waist circumference ≥80 cm for women are defined as abdominal obesity [20].

### 2.4. Assessment of Covariates

All participants were divided into 18–44 and 45–64 age groups, with three education levels (primary school and below, middle school, or high school and above). The yearly income was categorized into tertiles (low, medium, and high) in the analyses. We classified the smoking status as current or ever/never. We categorized alcohol drinking status as never drinking and drinking.

The area of residence (urban or rural) and the location of residence (northern or southern) were considered. Taking Qinling Mountains and Huaihe River as the dividing line, the investigated provinces are divided into southern and northern regions

Physical activity was based on the average number of working hours per week reported by participants: occupation, housework, leisure time, and transportation activities. The total physical activity was categorized into tertiles (low, medium, and high) in the analyses.

The urbanization index was divided into three groups (low, medium, and high) according to the urbanization index of subjects, which was calculated by 12 dimensions of the community level [21].

### 2.5. Statistical Analysis

Participants were divided into four groups according to the quartile of DED. Chi-square test (categorical variable) and one-way analysis of variance (ANOVA) or analysis of covariance (ANCOVA) (continuous variable) was used to test the differences in general characteristics of participants with different DED levels at baseline.

Due to the nested structure of the survey data, it violates the assumptions of data independence and homogeneity of variance in traditional statistical methods. A three-level (community level, individual level, observation level) mixed-effect linear regression model is used to estimate the relationship between DED and waist circumference, and three-level (community level, individual level, observation level) mixed-effect logistic regression model is used to evaluate the relationship between DED and abdominal obesity. Due to the differences in eating habits and physiology between different genders and the interaction between gender and DED, we conducted stratified analysis by gender. Four models were constructed. The results of the interaction between gender and dietary energy density are described in Tables S2 and S3 Model 1 includes only the survey year. Model 2 also adjusted for baseline age, urban and rural areas, and regions. Model 3 further adjusted income level, education level and urbanization index, physical activity, smoking status, alcohol consumption. Model 4 further adjusted for the energy proportion of protein, energy proportion of fat, and energy proportion of carbohydrate. In addition, we assessed the linear trend by assigning participants the median of the quartile of DED, and input this variable into the regression model as a continuous term.

All analyses were conducted using SAS (version 9.4 SAS Institute, Inc., Cary, NC, USA) and Stata version 15.0 (College Park, TX, USA). Inspection level α = 0.05.

## 3. Results

### 3.1. Baseline Characteristics by the Quartile of DED

Table 1 showed that a total of 6440 subjects were included in the baseline study, of whom 46.4% were male, with an average age of 39.7 ± 12.6 years, and 53.6% were female, with an average age of 39.2 ± 12.4 years. Among male and female subjects, there were significant differences between urban and rural, regions, and income levels among DED quartiles (*p* < 0.05). Among the male subjects, there was a significant difference in education level among different DED quartiles (*p* < 0.05). The physical activities of male and female subjects in the highest DED quartile were lower than those in other groups, and the waist circumference and the rates of abdominal obesity were higher than those in other groups. The total energy intake and the energy proportion of fat were higher in the highest DED quartiles than those of other groups. The energy proportion of protein and energy proportion of carbohydrates were lower than those of other groups.

### 3.2. Changes in DED and Waist Circumference in Males and Females from 1993 to 2018

**Figure 1:**
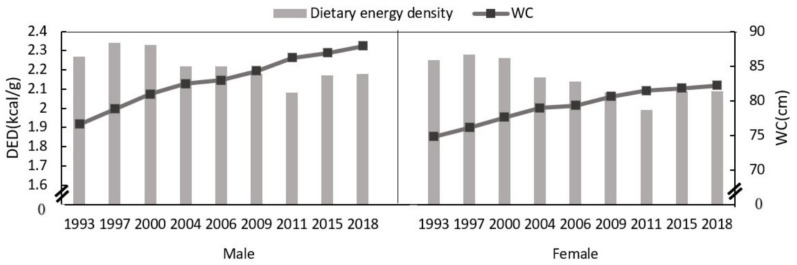
Changes in DED and waist circumference of male and female aged 18–64 years from 1993 to 2018.

From 1993 to 2018, the waist circumference of males increased from 76.6 cm in 1993 to 87.9 cm in 2018, and the waist circumference of females increased from 74.8 cm in 1993 to 82.2 cm in 2018. From 1993 to 2018, the DED of males decreased from 2.27 kcal/g in 1993 to 2.18 kcal/g in 2018, and the DED of females decreased from 2.25 kcal/g in 1993 to 2.09 kcal/g in 2018. (Figure 1).

### 3.3. Association between DED and Waist Circumference in Subjects Aged 18–64 from 1993 to 2018

Table 2 showed that, compared with the lowest DED quartile, the highest DED quartile was not associated with waist circumference in males. Compared with the lowest DED quartile, the highest DED quartile had a significant increase in waist circumference of 0.24 cm (95% CI: 0.39–1.09) after adjusting for all potential confounders.

### 3.4. Association between DED and Abdominal Obesity in Subjects Aged 18–64 from 1993 to 2018

Table 3 showed that the highest DED quartile had an increased risk of abdominal obesity, compared with the lowest DED quartile in females (OR = 1.16, 95% CI: 1.05, 1.29). No association was found between DED and abdominal obesity in males. 

## 4. Discussion

This study found that DED had a downward trend from 1993 to 2018, which was somewhat inconsistent with previous research. Research on adult residents aged 25–74 in the United States showed that the DED (including food and nutritional beverages) increased from 1.60 ± 0.01 kcal/g to 1.69 ± 0.02 kcal/g from 1971 to 2002 [22]. There was no significant trend in DED (including food and milk only) in the Scottish population from 2001 to 2009 [23]. The inconsistency of research results might be related to the difference in cooking methods and food choices between China and Western countries [24]. In China, boiling and steaming are mostly used for cereals and potatoes, while grinding and baking are mostly used in western countries. The decrease in DED of Chinese residents may be related to the change in dietary structure. In the past 30 years, the rapid development of China’s economy and food industry has caused significant changes in the dietary structure of Chinese residents. In recent years, the intake of cereals and potatoes has decreased, while the intake of foods rich in sugar, fat, refined carbohydrates, and animal foods has increased [25]. Studies have shown that from 1992 to 2010–2012, the proportion of Chinese residents’ energy intake from cereals decreased from 66.8% to 53.1%, and the proportion from animal food increased from 9.3% to 15.0% [26]. Cereals and potatoes, as a relatively economical food source for residents’ energy intake, account for a large proportion of adult residents’ dietary intake. This has had a significant impact on the dietary structure of residents, resulting in a downward trend in DED.

This study showed that DED was significantly associated with waist circumference and abdominal obesity in females. However, no association between DED and waist circumference and abdominal obesity was observed in males. There are still inconsistent results in domestic and foreign studies on the relationship between DED and waist circumference and abdominal obesity. The European Prospective Investigation into Cancer and Nutrition (EPIC) study of adults aged 20 to 78 years showed that DED was positively associated with changes in waist circumference [27]. NHANES results from 1999 to 2002 showed that the DED of American adults over 20 years old was positively correlated with waist circumference [28]. The results of a meta-analysis showed that there was no significant association between DED and the increased prevalence of central obesity [29]. These are not consistent with the results of this study. Inconsistent results may be related to differences in Chinese and Western diets. Besides this, inconsistent results may be related to inconsistencies in the methods used to investigate dietary data, with some studies using a food frequency method to collect meals, and some studies using three consecutive 24-h dietary recalls. In addition, different methods of calculating DED may also explain part of the reasons for the different results. However, a study in Japanese adults showed that DED was positively associated with abdominal obesity in females but not in males [30]. Yin Jun et al. found that in southwestern China, DED was positively correlated with waist circumference in females, while no correlation was found between DED and waist circumference in males [13]. These are consistent with the results of this study. The reason for DED having different effects on the occurrence of abdominal obesity in males and females may due to the differences in sex hormones between males and females [31]. On the other hand, nutritional knowledge and attitude may cause gender differences in dietary behavior [32,33].

How DED affects the development of abdominal obesity is still under investigation. It may be related to the influence of DED on energy intake. Research showed that adult residents were more likely to eat the same weight of food, so when eating high-energy-density food, it may lead to excessive energy intake [6]. Different dietary energy densities have great differences in the content of nutrients. The high content of fat and sugar in foods with high-energy-density may be related to the increased prevalence of abdominal obesity [34]. DED may affect energy intake by affecting satiety. Low DED may reduce energy intake by enhancing satiety [35]. Studies have shown that high-energy-density foods are tasty but not satisfying, while low-energy-density foods are satiating but less palatable. Food cost of different dietary energy densities was also one of the important factors influencing food choice. Research suggested that the price of high-energy-density food was lower than that of low energy density food [36]. However, the results of some studies showed that the cost of low DED was not higher than that of high DED, but the expenditure of high DED on takeout food was significantly higher than that of low DED [37].

The strength of our study was that we used longitudinal data from CHNS from 1993 to 2018 with a relatively large sample size. However, this study also had some limitations. First, CHNS used three consecutive 24-h dietary recalls to collect dietary information, which might not reflect the long-term dietary habits of the respondents. In addition, study subjects who chose a high-fat diet and people with abdominal obesity may consciously reduce or underreport food intake.

## 5. Conclusions

With the acceleration of China’s urbanization and the change in residents’ dietary habits, the DED of Chinese residents showed a downward trend from 1993 to 2018. The highest DED increased females’ waist circumference and abdominal obesity. However, no association between DED and waist circumference and abdominal obesity was observed in males. In the future, it is necessary to carry out further research on high DED and abdominal obesity, so as to provide a scientific basis for taking more effective measures to control abdominal obesity.

## Figures and Tables

**Table 1:** Baseline characteristics of participants by quartiles of DED in males and females.

Characteristics	Males				*p*	Females				*p*
	Q_1_^1^	Q_2_	Q_3_	Q_4_		Q_1_	Q_2_	Q_3_	Q_4_	
N	731	775	730	752		851	879	857	865	
DED (kcal/g) ^2^	1.7 ± 0.0	2.1 ± 0.0	2.4 ± 0.0	2.8 ± 0.0	<0.001	1.7 ± 0.0	2.1 ± 0.0	2.4 ± 0.0	2.8 ± 0.0	<0.001
Age (years)	39.9 ± 0.5	40.1 ± 0.5	39.2 ± 0.5	39.5 ± 0.5	0.544	39.1 ± 0.4	39.5 ± 0.4	39.1 ± 0.4	39.0 ± 0.4	0.814
Urban and rural (%)										
Urban	21.5	30.7	33.7	36.8	<0.001	23.7	31.0	34.2	34.8	<0.001
Rural	78.5	69.3	66.3	63.2		76.3	69.0	65.8	65.2	
Region (%)										
North	30.8	29.8	34.7	45.1	<0.001	29.7	32.3	37.7	46.8	<0.001
South	69.2	70.2	65.3	54.9		70.3	67.7	62.3	53.2	
Education level (%)										
Primary school and below	47.2	41.9	41.5	37.9	0.010	60.8	56.9	57.6	61.0	0.100
Middle school	33.7	34.2	36.6	40.0		26.4	26.2	25.8	25.9	
High school and above	19.1	23.9	21.9	22.1		12.8	16.9	16.6	13.1	
Income level (%)										
Low	36.4	28.1	30.4	37.1	0.001	33.0	29.7	32.9	39.1	0.005
Medium	32.7	34.5	36.4	32.2		32.3	34.2	34.0	30.3	
High	30.9	37.4	33.2	30.7		34.7	36.1	33.1	30.6	
Urbanicity index	44.4 ± 0.6	50.1 ± 0.6	50.7 ± 0.6	47.5 ± 0.6	<0.001	46.3 ± 0.6	49.2 ± 0.6	50.1 ± 0.6	46.8 ± 0.6	<0.001
Physical activity (MET hours/week)	381.9 ± 8.6	323.6 ± 7.6	324.4 ± 7.9	336.4 ± 8.2	<0.001	433.0 ± 9.1	392.6 ± 8.8	380.4 ± 8.9	392.5 ± 8.6	<0.001
Current smoker (%)	66.3	66.7	67.1	69.8	0.474	3.8	5.1	4.5	3.4	0.339
Alcohol consumption (%)	64.6	63.0	64.0	63.0	0.904	13.8	11.7	11.2	10.8	0.214
WC (cm)	75.9 ± 0.3	76.1 ± 0.3	76.8 ± 0.3	77.5 ± 0.3	0.001	74.2 ± 0.3	74.1 ± 0.3	75.1 ± 0.3	76.0 ± 0.3	<0.001
Abdominal obesity (%)	16.4	15.6	16.4	21.1	0.018	23.2	24.9	28.5	34.0	<0.001
Dietary intake										
Total energy(kcal/day)	2237.0 ± 2 7.8	2637.2 ± 20.7	2839.6 ± 24.3	2988.0 ± 23.3	<0.001	2066.9 ± 20.7	2310.6 ± 17.4	2445.6 ± 20.3	2649.4 ± 20.8	<0.001
The proportion of energy protein	13.6 ± 0.1	13.3 ± 0.1	12.9 ± 0.1	12.2 ± 0.1	<0.001	13.6 ± 0.1	13.2 ± 0.1	12.7 ± 0.1	12.2 ± 0.1	<0.001
The proportion of energy fat	23.0 ± 0.5	23.3 ± 0.4	25.5 ± 0.4	26.2 ± 0.4	<0.001	21.6 ± 0.4	22.8 ± 0.3	24.1 ± 0.4	25.7 ± 0.4	<0.001
The proportion of energy carbohydrate	63.4 ± 0.5	63.4 ± 0.4	61.5 ± 0.4	61.6 ± 0.4	<0.001	64.8 ± 0.4	64.0 ± 0.4	63.2 ± 0.4	62.1 ± 0.4	<0.001

^1^ Q = quartile, ^2^ Mean ± standard error. ANOVA or ANCOVA tests were used for continuous variables, and chi-square test was used for categorical variable. Adjusted by age for total energy, the energy proportion of protein, the energy proportion of fat, and the energy proportion of carbohydrate, and physical activity.

**Table 2:** Association between DED and waist circumference in subjects aged 18–64, CHNS (1993–2018) ^1^.

	DED				*p*-Trend ^3^
	Q_1_ ^2^	Q_2_	Q_3_	Q_4_	
Male					
Model 1 ^4^	0	0.18 (−0.03, 0.39)	0.07 (−0.14, 0.29)	−0.05 (−0.27, 0.17)	0.561
Model 2	0	0.21 (−0.01, 0.42)	0.11 (−0.11, 0.32)	−0.01 (−0.22, 0.21)	0.825
Model 3	0	0.21 (−0.01, 0.42)	0.10 (−0.11, 0.32)	−0.01 (−0.22, 0.22)	0.842
Model 4	0	0.23 (0.02, 0.45) *	0.14 (−0.08, 0.36)	0.01 (−0.22, 0.25)	0.984
Female					
Model 1	0	−0.02 (−0.22, 0.19)	0.09 (−0.11, 0.30)	0.29 (0.07, 0.50) **	0.004
Model 2	0	0.01 (−0.19, 0.21)	0.13 (−0.08, 0.33)	0.33 (0.12, 0.54) **	0.001
Model 3	0	−0.01 (−0.20, 0.20)	0.09 (−0.11, 0.29)	0.27 (0.06, 0.48) *	0.006
Model 4	0	−0.01 (−0.21, 0.20)	0.08 (−0.13, 0.29)	0.24 (0.01, 0.46) *	0.024

^1^ All of the models used three-level mixed-effects linear regression. ^2^ Q = quartile. ^3^ *p*-trend was calculated across the quartiles of DED, and the median value for each quartile was entered as a continuous term in the regression models. ^4^ Model 1 adjusted for surveyed year; model 2 adjusted for baseline-age, urban and rural, region on the basis of model 1; model 3 adjusted for income level, education level, urbanization index, physical activity, smoking status, alcohol consumption on the basis of model 2; model 4 adjusted for the energy proportion of protein, the energy proportion of fat, and the energy proportion of carbohydrate on the basis of model 3. * *p* < 0.05; ** *p* < 0.01.

**Table 3:** Association between DED and abdominal obesity in subjects aged 18–64, CHNS (1993–2018) ^1^.

	DED				*p*-Trend ^3^
	Q_1_^2^	Q_2_	Q_3_	Q_4_	
Male					
Model 1 ^4^	1.00	1.03 (0.93, 1.14)	1.02 (0.92, 1.13)	1.00 (0.90, 1.11)	0.986
Model 2	1.00	1.04 (0.94, 1.16)	1.04 (0.94, 1.15)	1.02 (0.92, 1.14)	0.677
Model 3	1.00	1.05 (0.95, 1.16)	1.05 (0.94, 1.16)	1.02 (0.93, 1.14)	0.593
Model 4	1.00	1.06 (0.96, 1.18)	1.07 (0.96, 1.19)	1.05 (0.93, 1.17)	0.397
Female					
Model 1	1.00	1.01 (0.92, 1.11)	1.05 (0.96, 1.16)	1.15 (1.04, 1.26) **	0.003
Model 2	1.00	1.04 (0.95, 1.14)	1.07 (0.98, 1.18)	1.18 (1.07, 1.29) **	0.003
Model 3	1.00	1.03 (0.94, 1.13)	1.06 (0.97, 1.16)	1.15 (1.05, 1.27) **	0.002
Model 4	1.00	1.04 (0.94, 1.14)	1.07 (0.97, 1.18)	1.16 (1.05, 1.29) **	0.003

^1^ All of the models used three-level mixed-effects logistic regression. ^2^ Q = quartile. ^3^ *p*-trend was calculated across the quartiles of DED, and the median value for each quartile was entered as a continuous term in the regression models. ^4^ Model 1 adjusted for surveyed year; model 2 adjusted for baseline-age, urban and rural, region on the basis of model 1; model 3 adjusted for income level, education level, urbanization index, physical activity, smoking status, alcohol consumption on the basis of model 2; model 4 adjusted for the energy proportion of protein, the energy proportion of fat, and the energy proportion of carbohydrate on the basis of model 3. ** *p* < 0.01.

## Data Availability

The datasets generated during and/or analyzed during the current study are available from the corresponding authors (C.S.) on reasonable request.

## Supplementary Materials

The following supporting information can be downloaded at: https://www.mdpi.com/article/10.3390/nu14102151/s1, Table S1: Grouping standard of DED. Table S2 Relationship between waist circumference and DED, gender and their interaction in Chinese subjects aged 18–64 (1993–2018). Table S3 Relationship between abdominal obesity and DED, gender and their interaction in Chinese subjects aged 18–64 (1993–2018).
