# Supplementary material for: Longitudinal Association of Dietary Energy Density with Abdominal Obesity among Chinese Adults from CHNS 1993–2018
Source: Nutrients. 2022 May 21;14(10):2151. doi: 10.3390/nu14102151 (PMC9143873; doi:10.3390/nu14102151)
Supplement: Supplementary file 1 [file nutrients-14-02151-s001.zip › nutrients-1682479-supplementary.pdf]

Table S1 Grouping standard of dietary energy density

	Males				Females			
	Q_1[1]	Q_2	Q_3	Q_4	Q_1	Q_2	Q_3	Q_4
1993	<2.01	2.01~2.26	2.27~2.53	≥2.54	<1.98	1.98~2.23	2.24~2.50	≥2.51
1997	<2.09	2.09~2.33	2.34~2.58	≥2.59	<2.02	2.02~2.26	2.27~2.53	≥2.54
2000	<2.06	2.06~2.30	2.31~2.58	≥2.59	<1.98	1.98~2.24	2.25~2.51	≥2.52
2004	<1.93	1.93~2.19	2.20~2.47	≥2.48	<1.86	1.86~2.13	2.14~2.42	≥2.43
2006	<1.94	1.94~2.20	2.21~2.48	≥2.49	<1.85	1.85~2.13	2.14~2.41	≥2.42
2009	<1.90	1.90~2.17	2.18~2.43	≥2.44	<1.79	1.79~2.05	2.06~2.34	≥2.35
2011	<1.76	1.76~2.04	2.05~2.36	≥2.37	<1.67	1.67~1.95	1.96~2.27	≥2.28
2015	<1.88	1.88~2.14	2.15~2.43	≥2.44	<1.78	1.78~2.06	2.07~2.36	≥2.37
2018	<1.88	1.88~2.15	2.16~2.45	≥2.46	<1.77	1.77~2.05	2.06~2.35	≥2.36

[1]Q = quartile.

Table S2 Relationship between waist circumference and DED, gender and their interaction in Chinese subjects aged 18 ~ 64 (1993-2018)

	b(95%CI)	P
DED		
Q1	0.00	
Q2	0.13(-0.10,0.37)	0.267
Q3	0.08(-0.15,0.32)	0.488
Q4	-0.07(-0.32,0.17)	0.548
Gender		
Male	0.00	
Female	-4.57(-4.86,-4.27)	<0.001
Gender * DED		
Gender *Q1	0.00	
Gender *Q2	-0.13(-0.45,0.19)	0.431
Gender *Q3	0.11(-0.22,0.43)	0.521
Gender *Q4	0.48(0.15,0.82)	0.004

The models were constructed using three-level mixed-effects linear regression with maximum likelihood estimation methods.

Q1-Q4 is the quartile grouping of DED.

Table S3 Relationship between abdominal obesity and DED, gender and their interaction in Chinese subjects aged 18 ~ 64 (1993-2018)

	OR(95%CI)	P
DED		
Q1	1.00	
Q2	1.02(0.93,1.12)	0.645
Q3	1.02(0.93,1.12)	0.668
Q4	0.99(0.90,1.08)	0.775
Gender		

Male	1.00	
Female	1.04(0.94,1.16)	0.414
Gender * DED		
Gender *Q1	1.00	
Gender *Q2	0.99(0.87,1.12)	0.890
Gender *Q3	1.05(0.92,1.19)	0.470
Gender *Q4	1.17(1.03,1.33)	0.017

The models were constructed using three-level mixed-effects logistic regression

Q1-Q4 is the quartile grouping of DED.
